# Repeated ambulance use is associated with chronic diseases - a population-based historic cohort study of patients’ symptoms and diagnoses

**DOI:** 10.1186/s13049-019-0624-4

**Published:** 2019-04-16

**Authors:** Morten Breinholt Søvsø, Torben Anders Kløjgaard, Poul Anders Hansen, Erika Frischknecht Christensen

**Affiliations:** 1 Centre for Prehospital and Emergency Research, Department of Clinical Medicine, Aalborg University, Søndre Skovvej 15, 9000 Aalborg, Denmark; 2 Emergency Medical Services, Aalborg, North Denmark Region Denmark

**Keywords:** Emergency medical services, Denmark, Ambulance, Emergency call, Repeated users, Frequent users, Diagnoses

## Abstract

**Background:**

There is a growing demand for emergency medical services (EMS) and patients are repeatedly transported by ambulance services. For many patients, especially those with chronic disease, there may be better ways of delivering care. We examined the symptom at time of emergency call and the hospital diagnosis for those ambulance users who repeatedly received an ambulance.

**Methods:**

Population-based historic cohort study of patients receiving an ambulance after an emergency call between 2011 and 2014: one-time users (i.e. one ambulance run in any 12 month period) were compared to two-time users (two runs in any 12 month period) and frequent users (>two runs). The presenting symptom according to the Danish Index for Emergency Care from the EMS calls and the hospital ICD-10 discharge diagnoses were obtained from patient records.

**Results:**

We included 52 533 patients (65 932 emergency ambulance runs). Repeated users constituted 16% of the patients (two-time users 11% and frequent users 5%) and one third of all ambulance runs. The symptoms showing the largest increase in frequency with increasing ambulance use were breathing difficulty (*N* = 3 905–15% were frequent users); seizure (*N* = 2 437–10% were frequent users), chest pain (*N* = 7 616–17% were frequent users), and alcohol intoxication (*N* = 1 998–5% were frequent users). The hospital diagnoses with a corresponding increase were respiratory diseases (*N* = 4 381) - 13% were frequent users), mental disorders (predominately abuse of alcohol) (*N* = 3 087–10% were frequent users) and neurological diseases (predominately epilepsy) (*N* = 2 207–6% were frequent users). 5% of one-time users, 12% of two-time users and 16% of frequent users had a Charlson Comorbidity Index > = 3.

**Conclusion:**

Repeated use of ambulance services was common and associated with chronic health problems such as chronic respiratory diseases, epilepsy, mental disorders with alcohol abuse and comorbidity. Alternative methods of caring for many of these patients should be considered.

**Trial registration:**

None.

**Electronic supplementary material:**

The online version of this article (10.1186/s13049-019-0624-4) contains supplementary material, which is available to authorized users.

## Introduction

Emergency Medical Services (EMS) experience a growing demand for their services [1, 2]. The reasons are not quite clear, but might be explained by demographics and disease patterns, with more elderly people [3] with more chronic diseases and consequently more comorbidity [4, 5]. On the other hand, health care system-factors such as less easy access to primary care [6], fewer hospital beds, shorter admissions and repeated use of emergency medical services [7] may play a role. A recent Danish study reported a 67% increase in the number of emergency ambulance runs to hospital from 2007 to 2014, and 25% of the patients had more than one ambulance run [8]. These growing numbers of hospital admissions have prompted the need to identify those patients that might be better managed in a different setting [9].

A systematic review from 2014 investigating frequent use of EMS [10] found that between 0.2 and 23% of ambulance users were repeated users, although this term was defined very differently across studies. The review also found that repeated users were more likely to suffer from medical conditions than trauma. However, 10 of the 18 included studies were emergency department-based and the review therefore suggested further research on repeated EMS users only. Like all other services in health care, the EMS has limited resources, and whenever ambulances are dispatched to one incident, they become unavailable to another. In order to explain the repeated use of ambulances, detailed knowledge is needed of those patients who repeatedly receive emergency ambulance runs to hospital.

Facilitated by the Danish patients’ unique civil registration number, which can link EMS data to health care registries containing the patients’ hospital diagnoses, this study compared the presenting symptoms at the emergency call to the EMS, hospital discharge diagnoses and comorbidity for repeated ambulance users with one-time users.

## Methods

### Study design

A population-based historic cohort study of patients to whom an ambulance was dispatched after a call to the Danish national emergency number 1–1-2 in the North Denmark Region. Symptoms at the time of the 1–1-2 call were retrieved and presented according to the criteria of the Danish Index for Emergency Care (Danish Index) [11]. Hospital discharge diagnoses according to the International Statistical Classification of Diseases and Related Health Problems, 10th Revision (ICD-10) [12] and patient demographics were retrieved from the regional Patient Administrative System (PAS) [13].

### Study setting

The North Denmark Region (580 000 inhabitants) as reported by Christensen et al. [2, 8]. In Denmark, EMS and ambulance services are tax-supported and equally accessible to all citizens. In every region when an Emergency Medical Coordination Centre receives a 1–1-2 medical emergency call a health care professional then assesses its urgency and severity. This is done according to the Danish Index [11], which contains 37 criteria corresponding to clinical signs, symptoms or incidents. The health care professional can also decide to end call by giving advice or referring the caller to a general practitioner. If needed, technical personnel then dispatch ambulances, helicopters and/or physician-staffed mobile units. An electronic pre-hospital medical record (amPHI™) has been used in all pre-hospital units in the region since April 2006. Logistic data on the ambulance runs i.e. “time stamps”, locations etc. are stored in separate logistics data bases.

### Study population and exclusions

Patients to whom an ambulance was dispatched after a 1–1-2 emergency call in the North Denmark Region during the period 1.1.2011–31.12.2014 were included in the study. All Danish citizens have a unique ten-digit civil registration number (CVR) that can be used to link to all data in any of the Danish national registries [14]: as this number is crucial to this study, patients who were not Danish citizens had to be excluded. Ambulance emergency runs where information on ambulance use in the previous 12 months was outside our study period were excluded (i.e. transfers before 1.1.2012).

### Patient classifications and outcomes

**Fig. 1:**
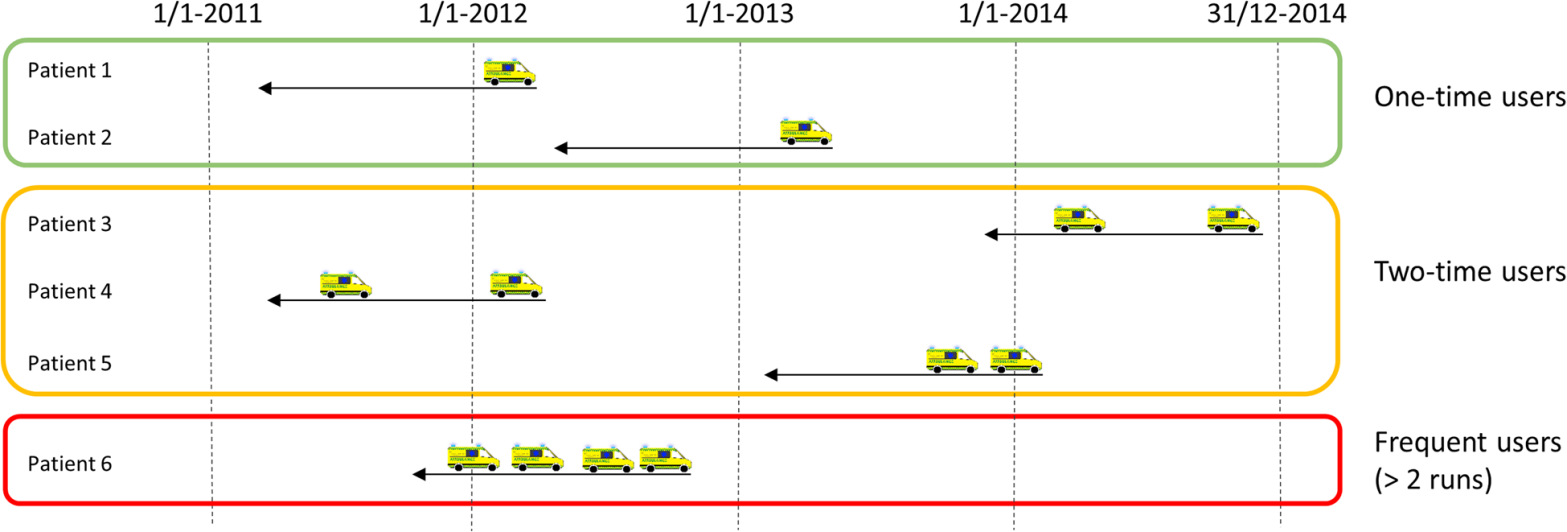
Definition of user groups. The three groups of users based on the identified ambulance runs in the study period

One-time users were defined as those patients with only one emergency ambulance run in any 12-month period, two-time users as those with two emergency runs in any 12-month period, and frequent users as those with more than two emergency runs in any 12-month period (Fig. 1). When referring to repeated users, this covers both two-time and frequent users.

The patient’s presenting symptom for each emergency ambulance run was defined by the Danish Index criteria, and the principal discharge diagnosis according to ICD-10 [12] was obtained from PAS for patients with ambulance runs to hospital. If the diagnosis was among the non-specific diagnoses (ICD-10 chapters Z and R) i.e. not related to a specific organ system or specific etiology (such as infection, cancer, injury, poisoning), we looked for a more specific diagnosis during the same hospital stay, and if found, this was used instead. Supplementary to the ICD-10 diagnoses at the chapter level, we investigated the most frequently used diagnoses at subcategory level, where particularly frequent diagnoses were reported separately. In addition, we investigated whether repeated users received the same diagnosis (at subcategory and chapter level) more than once. Not all ambulance runs received a symptom and/or a hospital diagnosis.

Comorbidity was assessed by the Charlson Comorbidity Index (CCI) [15, 16], based on diagnoses from the past five years retrieved from PAS. Patients were divided into four groups according to their CCI score: 0,1,2,> = 3.

### Statistical analysis

Data were anonymized for statistical analysis. We performed descriptive analysis reporting the distribution of one-time users and repeated users in numbers and frequencies among both patients and ambulance runs. Baseline characteristics (age, sex, comorbidity) were reported alongside. A logistic regression was used to check for significant differences in between one-time and repeated users. Similarly, we performed a logistic regression with random effects (since comorbidity can change between runs) to investigate differences of statistical significance in comorbidity.

Within in each of the user groups, we displayed the distribution of symptoms when calling EMS (i.e. Danish Index criteria) and the hospital diagnoses according to ICD-10 chapters as frequencies, thus, comparing one-time and repeated users. Analyses were performed on available symptoms and/or diagnosis. Logistic regressions with random effects were performed to calculate crude odds ratio (OR) for the symptoms showing the largest differences in proportions between one-time and repeated users, testing each symptom against all other symptom. Adjusting for comorbidity was not possible, as this group of patients included patients treated and left on scene without hospital contact i.e. without a current diagnosis, thus comorbidity is not available for this group. In addition, the distribution of age within each of the symptom groups differed too much to compare groups in terms of age (Additional file 1). Adjusting for age was possible by matching randomized age groups prior to the logistic regression. This would result in a comparison between matching age groups with many patients and almost no patients. Consequently, we would underestimate the significance of age for symptom groups with deviating age distributions. Therefore, we did not adjust for age in the analyses. Logistic regression analyses with random effects were performed for the hospital diagnoses showing the largest differences in proportions between one-time and repeated users, testing each diagnosis against all others. Both crude analyses and analyses adjusted for age, sex and comorbidity were performed. Subsequently, results were gathered in OR plots. We included all the patients´ ambulance runs in the analyses.

Statistical analyses were performed with Stata V.15.1 (Stata Corporation, College Station, Texas, USA).

## Results

**Fig. 2:**
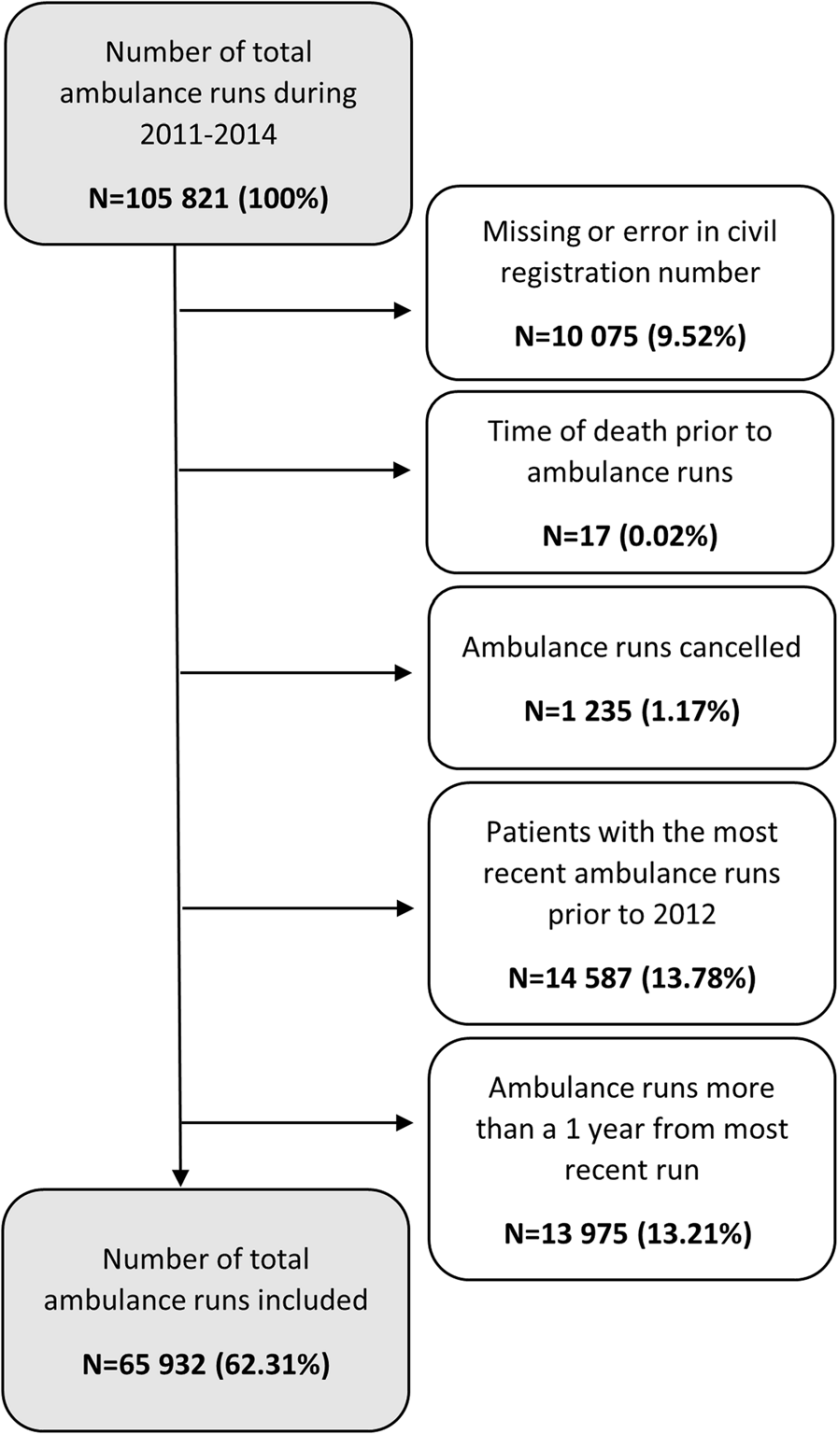
Flowchart of study inclusion process. Flowchart displaying the inclusion of the study population and exclusion criteria

Out of 105 821 dispatched ambulances in the North Denmark Region during the study period 39 889 (37.7%) were excluded. The reasons for exclusion are shown in Fig. 2, and the final study population was 65 932 emergency ambulance runs corresponding to 52 533 patients.

In 53 370 (81%) of the ambulance runs, the patients received a symptom according to the criteria in Danish Index. The ambulance runs resulted in hospital contacts in 60 459 (92%) of all runs.

Tables 1 and 2 shows the baseline characteristics of the study population and the distribution of ambulance runs.

**Table 1:** Population baseline characteristics

	All patients		One-time users		Two-time users		Frequent users	
Patients, n(%)	52 533	(100%)	44 099	(84%)	5 971	(11%)	2 463	(5%)
Male, n(%)	28 087	(53%)	23 378	(53%)^1^	3 300	(55%)	1 409	(57%)
Age, median (95% CI)	57	(56–58)	55	(54–56)	63	(62–64)	60	(59–61)
1–1-2 ambulance runs, n(%)	65 932	(100%)	44 099	(67%)	11 942	(18%)	9 891	(15%)

Baseline characteristics of the included patients for each user group

^1^Significantly different from repeated users: crude OR (95%CI) 1.10 (1.04–1.16) for two-time users and 1.18 (1.09–1.29) for frequent users

**Table 2:** Comorbidity

	All patients		One-time users		Two-time users		Frequent users	
Charlson Comorbidity Index^a^								
0	40 184	(61%)	29 987	(68%)	6 002	(50%)	4 195	(43%)
1	9 421	(14%)	5 173	(12%)	2 184	(18%)	2 064	(21%)
2	5 555	(9%)	3 033	(7%)	1 300	(11%)	1 222	(12%)
3+	5 299	(8%)	2 301	(5%)	1 414	(12%)	1 584	(16%)
Not brought to hospital	5 473	(8%)	3 605	(8%)	1 042	(9%)	826	(8%)

Ambulance runs for each of the user groups and comorbidity (^a^ based on ambulance runs with hospital contacts)

The final study population had a median age of 57 years, 47% were female, and repeated users were older than one-time users (Table 1). Males were significantly more likely to be repeated users..

In addition, the patients with any degree of comorbidity had higher odds of repeated ambulance use.; crude OR (95%CI) 2.25 (2.13–2.37) for two-time users and 3.10 (2.87–3.34) for frequent users compared to one-time users.

### Symptoms when calling EMS

**Fig. 3:**
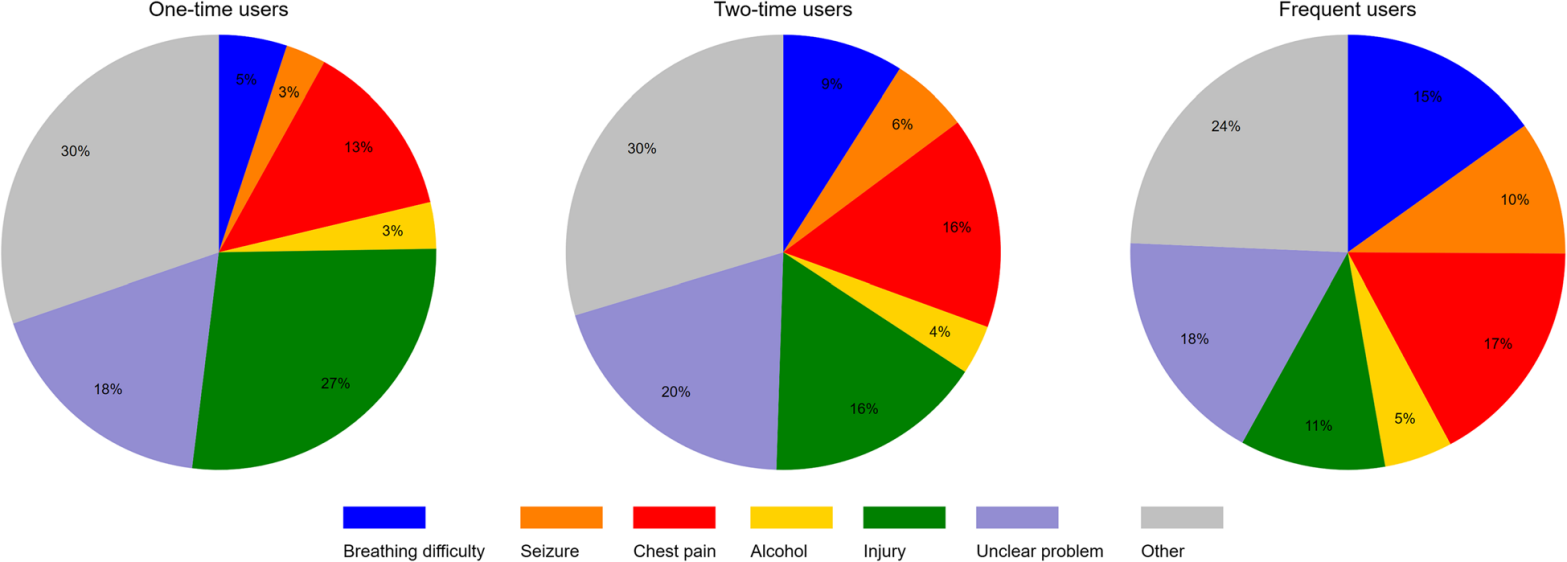
Distribution of symptoms when calling EMS among user groups. Distribution of symptoms assigned when calling EMS (*N* = 53 370 runs). ‘Injury’ is a collapsed category covering the three symptoms ‘wounds’, ‘accidents’ and ‘traffic accidents’

The symptoms that became more common with increased ambulance use were breathing difficulties, seizures, chest pain, and alcohol intoxication. Injuries, on the other hand, were more common among one-time users (Fig. 3).

**Fig. 4:**
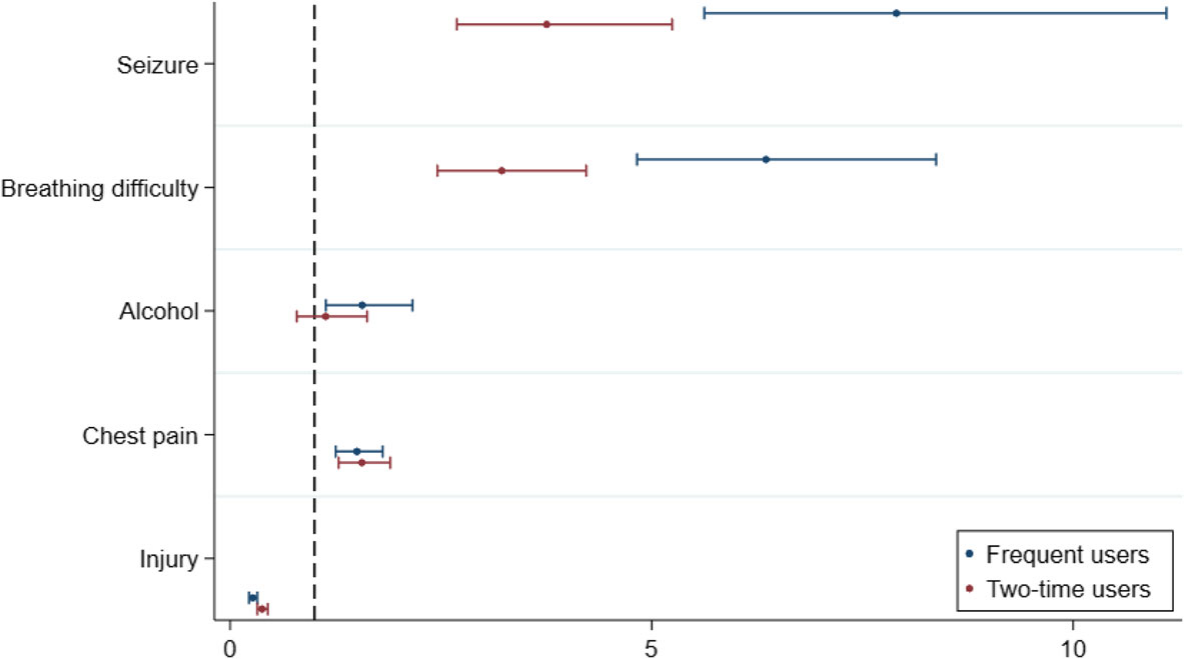
Association between user groups and symptoms. Crude odds ratio plot of the association between selected symptoms when calling EMS and ambulance use. The figure consists of several logistic regressions testing each symptom against all other symptoms. The dots represent the OR and the whiskers are the 95%CIs

Patients with difficulty breathing, seizures and chest pain at the time of the EMS call had significantly larger odds ratios for repeated ambulance use (Fig. 4) compared to all other symptoms. This was also the case for alcohol intoxication, which showed a significantly larger odds ratio for frequent ambulance use only.

On the other hand, injuries (including minor injuries, traffic accidents and other accidents) reduced the odds for repeated use significantly.

### Hospital diagnoses

Table 3 shows the top-10 distribution of diagnoses (ICD-10 chapters) for patients brought to hospital.

**Table 3:** Distribution of diagnosis at ICD-10 chapter level

	One-time users		Two-time users		Frequent users		Total	
Injuries and poisoning	13 510	(33%)	2 376	(22%)	1 382	(15%)	17 268	(29%)
Other factors	6 608	(16%)	1 670	(15%)	1 462	(16%)	9 740	(16%)
Symptoms and signs	5 386	(13%)	1 528	(14%)	1 257	(14%)	8 171	(14%)
Circulatory diseases	4 470	(11%)	1 509	(14%)	1 109	(12%)	7 088	(12%)
Respiratory diseases^a^	2 194	(5%)	987	(9%)	1 200	(13%)	4 381	(7%)
Mental disorders^a^	1 584	(4%)	611	(6%)	892	(10%)	3 087	(5%)
Digestive diseases	1 590	(4%)	515	(5%)	385	(4%)	2 490	(4%)
Neurological diseases^a^	1 110	(3%)	508	(5%)	589	(6%)	2 207	(4%)
Endocrine diseases	730	(2%)	324	(3%)	291	(3%)	1 345	(2%)
Genitourinary diseases	762	(2%)	234	(2%)	135	(1%)	1 131	(2%)
All remaining diagnoses	2 550	(6%)	638	(6%)	363	(4%)	3 551	(6%)
Total	40 494	(100%)	10 900	(100%)	9 065	(100%)	60 459	(100%)

Top-10 ICD-10 diagnoses at chapter level received at hospital and their distribution within the user groups

^a^Chapters with the largest difference in proportions of diagnoses between user groups

The ICD-10-chapters respiratory diseases, mental disorders and neurological diseases appeared in larger proportions among repeated users. Moreover, each of the chapters were dominated by one subcategory diagnosis; other chronic obstructive pulmonary disease (J44) 23% (one-time), 36% (two-time), 57% (frequent), mental and behavioural disorders due to abuse of alcohol (F10) 65, 63, 67%, epilepsy (G40) 35, 56, 75%.

Injuries/poisoning were more frequent among one-time users than repeated users and no single diagnosis dominated this chapter. Two-time users received the exact same subcategory diagnosis in 8% of the ambulance runs and a diagnosis within the same ICD-10 chapter in 19% of the runs. Ambulance runs with frequent user resulted in the same subcategory diagnosis twice in 14% of the runs and in 23% of the runs, they received a diagnosis within the same ICD-10 chapter twice.

**Fig. 5:**
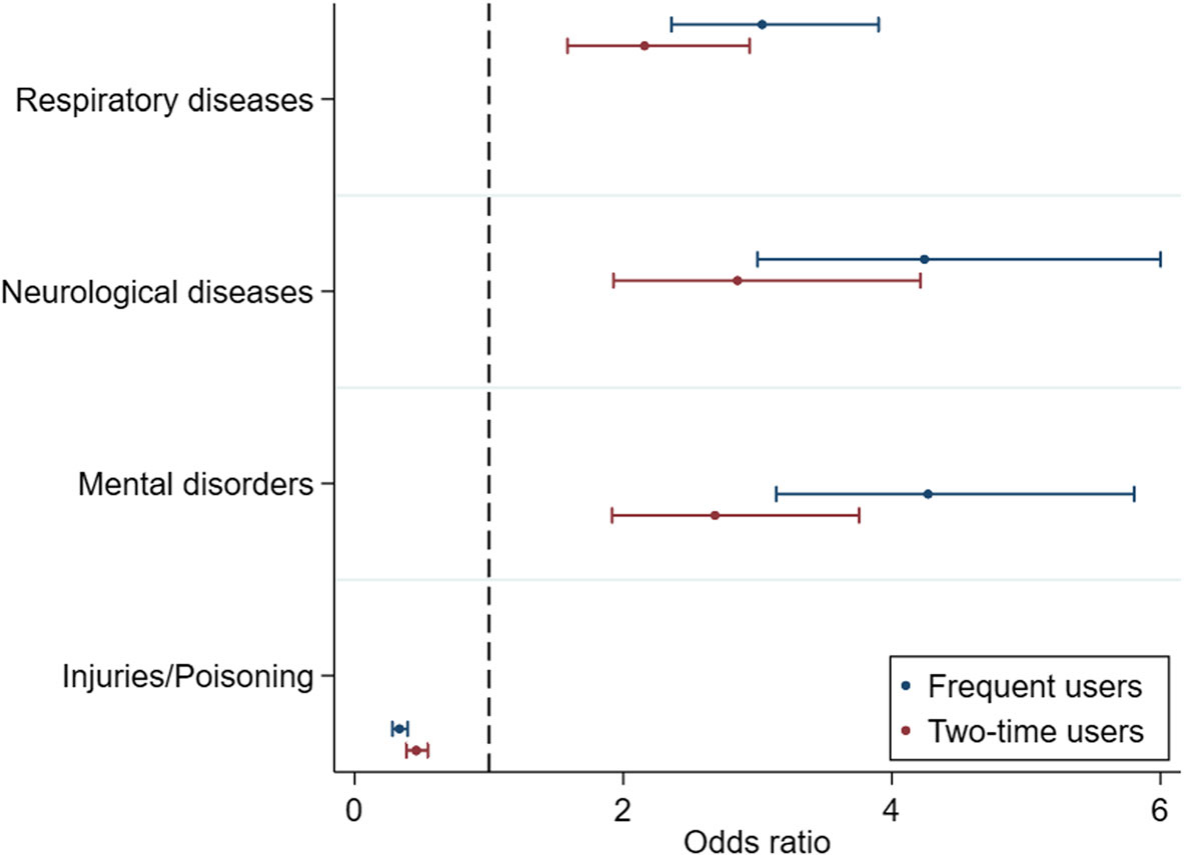
Association between user groups and hospital diagnoses. Odds ratio plot of the association between selected hospital diagnoses and ambulance use. Adjusted for age, gender and comorbidity. The figure consists of several logistic regressions testing each hospital diagnosis against all other diagnoses. The dots represent the OR and the whiskers are the 95%CIs

As illustrated in Fig. 5, for patients with a diagnosis of respiratory diseases, mental disorders and/or neurological diseases there was an increased likelihood of repeated ambulance use. The opposite was found for patients with a diagnosis of injuries/poisoning, who all had a decreasing odds ratio of repeated ambulance use.

## Discussion

This population-based historic cohort study found that one in three emergency ambulance runs were with repeated users. Furthermore, repeated ambulance users in the North Denmark Region constituted 16% of all pre-hospital patients. In addition, only 5% of patients had frequent ambulance use, but were responsible for 15% of all ambulance runs. Male patients, older patients, or patients with comorbidity were more likely to use ambulances repeatedly, as were patients with symptoms related to breathing difficulties, seizures and chest pain. Patients with the symptom alcohol intoxication at the time of the EMS call were also likely to be frequent users of emergency ambulances, and repeated ambulance users were more likely to have a discharge diagnosis of respiratory diseases, mental disorders (the majority due to alcohol abuse) and neurological diseases (predominately epilepsy).

### Other studies

A systematic review by Scott et al. [10] with 18 studies concluded that there is very little research on the characteristics of repeated EMS users, albeit they are more likely to suffer from medical conditions than trauma, as found in this study. The extent of repeated use differed a lot in the included studies – most likely because different definitions of repeated use were used.

Other studies have also found repeated calls to the EMS for help to be associated with chronic illness or behavioral health problems [6] including alcohol or substance abuse [17, 18]. Knowlton et al. (USA) [19] found that frequent users (six or more ambulance runs during their 23 months study) constituted 1.5% of the population and were more often male, African-American, older than 35 years, but with fewer frequent users in the age group above 75 years. They also found that at incident-level respiratory problems, seizures, and behavioral health problems were significantly more common among frequent users. Similar findings are also seen in a study by Chi et al. (Taiwan) [20], which investigated reasons for the ambulance runs for repeat users (two-three runs) and frequent users (more than three runs) and compared them to one-time users. The extent of repeated/frequent use was 7.3% and for this group, they found shortness of breath, dizziness/syncope/headache and seizure to occur at higher proportions than for one-time users.

Despite differences in study data quality and methodology, some of the characteristics of repeated ambulance users are clear: they often have behavioral or alcohol-related health problems, respiratory symptoms and/or illnesses, or seizures/epilepsy. These conditions are often chronic in nature and therefore these patients can be expected to experience acute exacerbations leading to health care contacts and repeated ambulance use. The results of the current study are expected to be applicable to countries with similar prehospital setups and populations such as other Scandinavian and certain European countries.

### Strengths and limitations

The unique CVR number for each patient is a major strength in our study as it allows us to perform extensive registry-linkage including symptoms and hospital diagnoses. In addition, the CVR number also allows us to determine repeated use with very high certainty.

Another major strength is the population-based design. Consequently, all contacts to EMS in the region are included, minimizing cohort selection bias and resulting in a large cohort.

We chose to describe the patients’ symptoms at the EMS call and the hospital diagnoses using ICD-10 chapters, which is s a major strength as it provides an overview of the entire care pathway from call to hospital.

Missing data is a limitation. We excluded ambulance runs without valid CVR number. Information on symptom was not available for all patients, and for patients not brought to hospital we had no diagnosis. This implies a risk of selection bias, but we cannot tell in which direction. However, due to the large number of included patients, the possible impact on the results would be minor.

Comorbidity was reported using Charlson Comorbidity Index score. However, we do not know to what extent patient contacts to EMS were due to the same preexisting chronic disease.

The data used in the current study is from 2011 to 2014, which limits the applicability to some extent, as recent changes in the pattern of repeated ambulance use will not be reflected in the study.

## Conclusions

In this population-based historic cohort study, we found that patients calling for an ambulance more than once in a 12-months period were frequent.

Furthermore, patients with symptoms such as breathing difficulties, seizures, chest pain and alcohol intoxication were more likely to be repeated users. There was good correspondence between the majority of these symptoms and the hospital diagnoses patients received, indicating that hospital contacts due to chronic diseases such as chronic obstructive pulmonary disease, epilepsy/seizures and alcohol abuse are associated with frequent and repeated use of ambulances.

In future studies, it would be important to investigate to which extent the patients are acutely and severely ill, and to which extent ambulance use is due to existing disease by comparing known patient comorbidity diagnoses and discharge diagnoses.

This study contributes with information on repeated ambulance users’ characteristics, which could interest health care planners and policy makers in the development of alternative, perhaps preventive, interventions for this group of patients.

## Additional file

Additional file 1: Age distribution among symptom groups. Histograms showing the distribution of age as percentage among the included symptom groups. (TIF 1025 kb)

## Abbreviations

CCI: Charlson Comorbidity Index
CI: Confidence interval
CVR: Danish civil registration system
Danish Index: Danish Index for Emergency Care
EMS: Emergency medical services
ICD-10: International Statistical Classification of Diseases and Related Health Problems 10th Revision
OR: Odds ratio
PAS: Patient Administrative System
