# Supplementary figures and images for: Repeated ambulance use is associated with chronic diseases - a population-based historic cohort study of patients’ symptoms and diagnoses
Source: Scand J Trauma Resusc Emerg Med. 2019 Apr 16;27:46. doi: 10.1186/s13049-019-0624-4 (PMC6469091; doi:10.1186/s13049-019-0624-4)

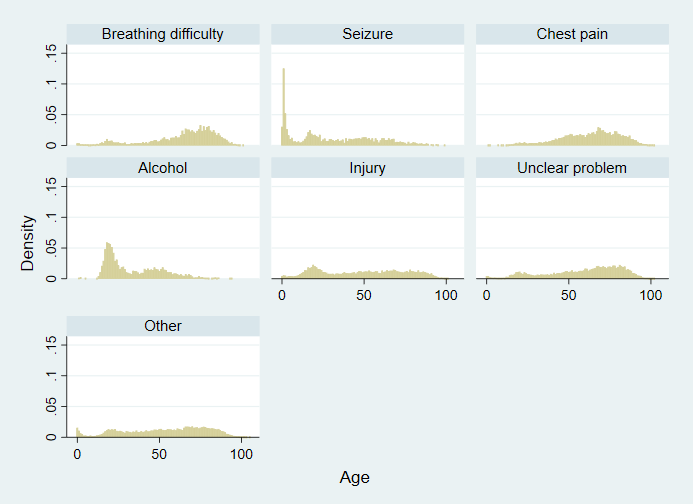

Supplement: Supplementary file 1 — Age distribution among symptom groups. Histograms showing the distribution of age as percentage among the included symptom groups. (TIF 1025 kb) [file 13049_2019_624_MOESM1_ESM.tif]
